# Stratifying prognosis in heart failure patients with reduced ejection fraction and atrial cardiomyopathy

**DOI:** 10.1093/eschf/xvag144

**Published:** 2026-05-21

**Authors:** Jung-Chi Hsu, Umbreen Nadeem, Khalid Kazi, Chris Hayward, Lan Mu, Wing Tak Wong, Gregory Y H Lip, Mark C Petrie, Dipak Kotecha, Alan John Camm, Jianhua Wu, Gary Tse, Chris P Gale, Ramesh Nadarajah

**Affiliations:** Division of Cardiology, Department of Internal Medicine, National Taiwan University Jinshan Branch, New Taipei City, Taiwan; Division of Cardiology, Department of Internal Medicine, National Taiwan University College of Medicine and Hospital, Taipei, Taiwan; Leeds Institute for Data Analytics, University of Leeds, Clarendon Way, Leeds LS2 9NL, UK; Leeds Institute for Cardiovascular and Metabolic Medicine, University of Leeds, 6 Clarendon Way, Leeds LS2 9DA, UK; Leeds Institute for Data Analytics, University of Leeds, Clarendon Way, Leeds LS2 9NL, UK; Leeds Institute for Cardiovascular and Metabolic Medicine, University of Leeds, 6 Clarendon Way, Leeds LS2 9DA, UK; Leeds Institute for Data Analytics, University of Leeds, Clarendon Way, Leeds LS2 9NL, UK; Leeds Institute for Cardiovascular and Metabolic Medicine, University of Leeds, 6 Clarendon Way, Leeds LS2 9DA, UK; Wolfson Institute of Population Health, Queen Mary University of London, London, UK; School of Life Sciences, Chinese University of Hong Kong, Hong Kong, China; Liverpool Centre for Cardiovascular Science at University of Liverpool, Liverpool John Moores University and Liverpool Heart & Chest Hospital, Liverpool, UK; Danish Center for Health Services Research, Department of Clinical Medicine, Aalborg University, Aalborg, Denmark; Department of Cardiology, Lipidology and Internal Medicine with Intensive Coronary Care Unit, Medical University of Bialystok, Bialystok, Poland; Institute of Cardiovascular and Medical Sciences, University of Glasgow, Glasgow, UK; Department of Cardiovascular Sciences, University of Birmingham, Edgbaston, Birmingham, UK; NIHR Birmingham Biomedical Research Centre, University Hospitals Birmingham NHS Trust, Birmingham, UK; Julius Center, University Medical Center Utrecht, Utrecht, The Netherlands; Molecular and Clinical Sciences Research Institute, St George’s University of London, London, UK; School of Life Sciences, Chinese University of Hong Kong, Hong Kong, China; School of Life Sciences, Chinese University of Hong Kong, Hong Kong, China; Diabetes Research Unit, Cardiovascular Analytics Group, Hong Kong, China; School of Nursing and Health Sciences, Hong Kong Metropolitan University, Hong Kong, China; Tianjin Key Laboratory of Ionic-Molecular Function of Cardiovascular Disease, Department of Cardiology, Tianjin Institute of Cardiology, Second Hospital of Tianjin Medical University, Tianjin, China; Leeds Institute for Data Analytics, University of Leeds, Clarendon Way, Leeds LS2 9NL, UK; Leeds Institute for Cardiovascular and Metabolic Medicine, University of Leeds, 6 Clarendon Way, Leeds LS2 9DA, UK; Department of Cardiology, Leeds Teaching Hospitals NHS Trust, Leeds General Infirmary, Great George Street, Leeds LS1 3EX, UK; Leeds Institute for Data Analytics, University of Leeds, Clarendon Way, Leeds LS2 9NL, UK; Leeds Institute for Cardiovascular and Metabolic Medicine, University of Leeds, 6 Clarendon Way, Leeds LS2 9DA, UK; Department of Cardiology, Leeds Teaching Hospitals NHS Trust, Leeds General Infirmary, Great George Street, Leeds LS1 3EX, UK

**Keywords:** Atrial cardiomyopathy, Heart failure with reduced ejection fraction, Prediction, Prognosis, FIND-AF

## Abstract

**Aims:**

Co-existence of atrial cardiomyopathy (AtCM) and heart failure with reduced ejection fraction (HFrEF) has prognostic implications. We aimed to quantify the association of a health records data biomarker, FIND-AF, with outcomes in patients with HFrEF and AtCM without atrial fibrillation (AF).

**Methods:**

Patients with HFrEF and AtCM without AF at baseline were identified from the ECG-HF registry (2010–6) and linked to the territory-wide Clinical Data Analysis and Reporting System. AtCM was operationalized using a pragmatic multi-domain framework based on the 2025 clinical consensus statement of the Heart Failure Association of the ESC, using electrocardiographic and echocardiographic parameters. Patients were stratified into high and low risk by sex-specific FIND-AF thresholds. The primary outcome was a composite of incident AF, ischaemic stroke, and all-cause mortality. All patients were followed until 31 December 2019.

**Results:**

We included 164 patients (mean age 62.3 ± 13.0 years, 73.8% men). The primary outcome occurred in 23/32 (71.9%) high-risk vs 67/132 (50.8%) low-risk patients (OR 2.48, 95% CI 1.07–5.76; *P* = .035). In the model including both FIND-AF risk group and AtCM severity, high FIND-AF risk remained associated with the composite outcome (adjusted OR 2.70, 95% CI 1.14–6.40; *P* = .024). Compared with CHA_2_DS_2_-VASc, FIND-AF showed superior discrimination for the composite outcome (AUC .735 vs .641; DeLong *P* = .007) and ischaemic stroke (AUC .700 vs .575; DeLong *P* = .008).

**Conclusions:**

High FIND-AF risk is associated with adverse prognosis in a cohort of patients with HFrEF and AtCM but without baseline AF. These exploratory data require external validation and a prospective study.

## Graphical Abstract

**Graphical Abstract:**
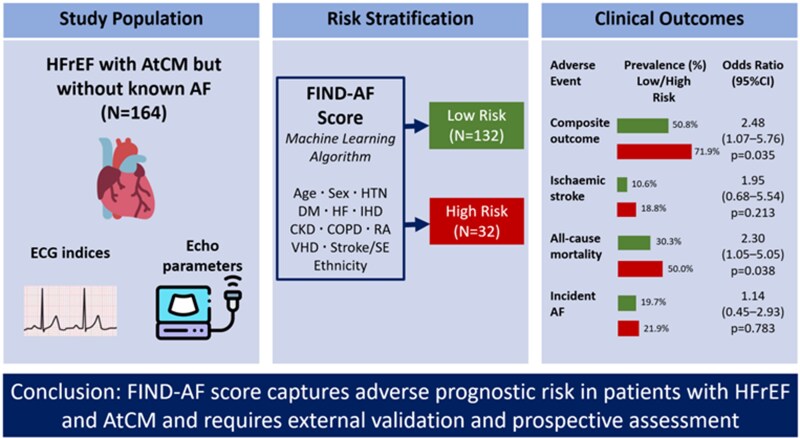

## Introduction

Atrial cardiomyopathy (AtCM) in patients with heart failure with reduced ejection fraction (HFrEF) is associated with increased risk of death and stroke.^1–3^ The Heart Failure Association (HFA) of the European Society of Cardiology identifies AtCM as a specific clinical entity and a potentially actionable target before the onset of atrial fibrillation (AF).^4^ At present, there is no way of stratifying risk in patients with HFrEF and atrial cardiomyopathy.

Future Innovations in Novel Detection of Atrial Fibrillation (FIND-AF) is a data biomarker that was developed using supervised machine-learning methods applied to routinely collected clinical data to predict incident AF in those without diagnosed AF.^5–7^ It may be applied to electronic health records or used as a hand-held calculator.^8^ Elevated FIND-AF risk in patients with HFrEF, but without AF, has been shown to be associated with adverse cardiac magnetic resonance (CMR) imaging characteristics and adverse clinical outcomes.^9^

This study aimed to quantify associations of FIND-AF risk with outcomes in patients with HFrEF and AtCM without AF. We hypothesized that high FIND-AF risk would be associated with increased incidence of adverse events in this patient group.

## Methods

### Study population

We analysed data from the ECG-HF registry, a prospective heart failure cohort established at the Joint Chinese University of Hong Kong. The registry enrolled adults hospitalized for heart failure at a single tertiary centre between 1 January 2010 and 31 December 2016, and the cohort has been previously described in detail in earlier publications.^10,11^ Patients were eligible for inclusion if they had a left ventricular ejection fraction ≤40%, no documented AF at baseline on ECG or diagnostic code in their electronic health record for AF, and at least one positive AtCM domain. Patients with documented prior AF in available diagnostic-code history were excluded when such information was available.

Baseline demographic, clinical, laboratory, electrocardiographic, and echocardiographic data were systematically collected. Deterministic linkage for clinical outcomes was performed using encrypted unique patient identifiers through the Clinical Data Analysis and Reporting System (CDARS), a territory-wide population database that integrates longitudinal medical records, diagnoses, laboratory results, prescriptions, and mortality information across all public hospitals in Hong Kong. CDARS has previously been shown to have good coding accuracy and data completeness.^12,13^

The study was approved by the Joint Chinese University of Hong Kong–New Territories East Cluster Clinical Research Ethics Committee. Additional ethical approval for secondary data analysis was obtained from the Institutional Review Board of National Taiwan University Hospital (202410100RINA).

### FIND-AF score and risk stratification

FIND-AF is a supervised machine learning algorithm (random forest) trained on routinely collected electronic health record data to predict incident AF within the next 6 months in patients aged ≥30 years.^6,7^ The final model includes the following 12 variables: age, sex, ethnicity (white or other), diabetes mellitus, heart failure, hypertension, stroke or systemic embolism, ischaemic heart disease, chronic obstructive pulmonary disease, valvular heart disease, chronic kidney disease, and rheumatoid arthritis.

For this study, the pretrained FIND-AF model was applied to the Hong Kong HFrEF registry to generate individual risk probabilities. Sex-specific thresholds (.0224 for men and .0350 for women) were used to define high-risk and low-risk categories, based on the thresholds associated with adverse prognosis and atrial dilatation in our previous work in a UK cohort.^9^ The overall and sex-stratified distribution of FIND-AF scores in the analytic cohort is shown in Supplementary Figure S1.

### Electrocardiographic assessment

A standard 12-lead electrocardiogram obtained at baseline was used for the extraction of atrial conduction and P-wave indices. Variables included P-wave duration, P-wave dispersion, P-wave terminal force in lead V1 (continuous and categorical), inter-atrial block (none, partial, advanced), and PR interval.

### Echocardiographic assessment

Two-dimensional transthoracic echocardiograms were performed at baseline using standardized clinical protocols. Measurements included left atrial diameter, left ventricular ejection fraction, left ventricular longitudinal strain, and left atrial strain components (reservoir, conduit, and contractile phases). Strain measures were averaged across apical views following the methodology established in prior reports from this registry.^10,11^ The echocardiography studies were conducted by qualified physicians or nurses with echocardiography training and checked by a cardiologist.

### Assessment of atrial cardiomyopathy

Atrial cardiomyopathy was characterized using a pragmatic multi-domain framework based on the 2025 clinical consensus statement of the Heart Failure Association of the ESC on atrial cardiomyopathy.^4^

Structural remodelling was defined by sex-specific left atrial enlargement using anteroposterior left atrial diameter ≥4.0 cm in men and ≥3.8 cm in women. These thresholds correspond to the sex-specific upper reference limits for LA anteroposterior diameter in the ASE/EACVI chamber quantification recommendations.^14^ Because LA volume was not consistently available in the source registry, LA anteroposterior diameter was used as the clinically accessible structural marker. Functional remodelling was defined as left atrial reservoir strain <15% in the main analysis, selected as a conservative HFrEF-specific threshold based on prior prognostic data showing that impaired left atrial reservoir function predicts adverse outcomes in HFrEF and that the median peak atrial longitudinal strain in this population was ∼15.5%.^1^ Electrical remodelling was identified using the HFA/ESC AtCM P-wave score: advanced inter-atrial block = 2 points; partial inter-atrial block, P-wave duration ≥120 ms, or P-wave terminal force in V1 ≥ 40 ms·mm = 1 point each. Because patients with baseline AF were excluded, the electrical domain was considered positive if the P-wave score was ≥1. Total AtCM scores of 1, 2, and ≥3 were defined as mild, moderate, and severe AtCM, respectively. An example of AtCM scoring is provided in Supplementary Table S1.

### Outcomes

The primary outcome was a composite of incident AF, ischaemic stroke, and all-cause mortality. Secondary outcomes included each component of the primary outcome. All clinical events were identified through CDARS. Patients were followed from baseline until 31 December 2019.

### Statistical analysis

Continuous variables are presented as mean ± standard deviation (SD) or median (interquartile range, IQR), as appropriate, and were compared using Student’s *t*-tests or Wilcoxon rank-sum tests. Categorical variables are presented as counts (percentages) and were compared using *χ*^2^ tests.

Univariable linear regression was performed to assess the association of FIND-AF score with electrocardiographic and echocardiographic parameters. Given the right-skewed distribution of the FIND-AF score, a natural logarithmic transformation was used for linear regression analyses.

Logistic regression models were used to assess the association between high vs low FIND-AF risk and clinical outcomes. Three progressively adjusted models were specified: Model 1, unadjusted; Model 2, adjusted for AtCM severity as a three-level factor; and Model 3, additionally adjusted for age and sex. For standard logistic regression models, odds ratios are presented with Wald 95% confidence intervals. Because age adjustment produced complete or near-complete separation in standard logistic regression, Model 3 was fitted using Firth’s penalized logistic regression.

Sensitivity analyses were performed using the 2025 clinical consensus statement of the Heart Failure Association of the ESC functional AtCM criterion of LA reservoir strain <23% and after excluding patients receiving anticoagulant therapy at baseline.^4^

Receiver operating characteristic (ROC) curves were used to compare the continuous FIND-AF score with CHA_2_DS_2_-VASc, and AUCs were compared using the DeLong test. Statistical significance was defined as a two-sided *P*-value <.05. Analyses were conducted using SPSS version 26.0 and R software version 4.3.

## Results

### Cohort derivation and atrial cardiomyopathy distribution

Of 312 patients with linked data and XML ECG recordings, 72 with baseline AF were excluded, 7 patients were excluded due to a left ventricular ejection fraction >40%, and 69 patients were excluded due to the absence of AtCM, yielding a final analytic cohort of 164 HFrEF patients with AtCM and no baseline AF (Supplementary Figure S2). Of the 164 patients, 32 (19.5%) were classified as high FIND-AF risk. High-risk patients were older (77.0 ± 7.7 vs 58.7 ± 11.4 years; *P* < .001) and had a higher neutrophil-to-lymphocyte ratio (*P* = .024) (*Table 1*). Most patients had severe AtCM (126 patients, 76.8%), followed by moderate (23 patients, 14.0%) and mild (15 patients, 9.1%) (Supplementary Figure S3). Positivity of the structural, functional, and electrical AtCM domains, stratified by FIND-AF risk category, is shown in Supplementary Figure S4.

**Table 1:** Baseline characteristics stratified by FIND-AF risk categories in HFrEF patients with atrial cardiomyopathy

Variable	Overall (*N* = 164)	Low FIND-AF (*N* = 132)	High FIND-AF (*N* = 32)	*P*-value
Age, years	62.30 ± 12.96	58.74 ± 11.37	77.00 ± 7.72	<.001
Male sex, *n* (%)	121 (73.8)	100 (75.8)	21 (65.6)	.345
Hypertension, *n* (%)	96 (59.6)	75 (58.1)	21 (65.6)	.568
Diabetes mellitus, *n* (%)	67 (40.9)	54 (40.9)	13 (40.6)	1.000
Hypercholesterolaemia, *n* (%)	61 (38.4)	49 (38.3)	12 (38.7)	1.000
Ischaemic heart disease, *n* (%)	97 (61.0)	73 (57.0)	24 (77.4)	.060
Smoking, *n* (%)	85 (53.5)	68 (53.1)	17 (54.8)	1.000
Chronic kidney disease, *n* (%)	48 (29.6)	42 (32.1)	6 (19.4)	.240
ICD implanted, *n* (%)	13 (8.0)	12 (9.2)	1 (3.1)	.438
CRT device, *n* (%)	28 (17.3)	23 (17.7)	5 (15.6)	.987
Neutrophil-to-lymphocyte ratio	4.21 [2.47–6.80]	4.16 [2.29–6.40]	5.18 [3.53–10.30]	.024
Serum albumin, g/L	34.34 ± 6.62	34.40 ± 6.64	34.08 ± 6.64	.824
Serum sodium, mmol/L	139.21 ± 4.45	138.90 ± 4.27	140.67 ± 5.05	.072
Serum potassium, mmol/L	4.08 ± .73	4.09 ± .78	4.05 ± .47	.799
Urea, mmol/L	8.77 ± 5.86	8.96 ± 6.35	7.98 ± 3.14	.400
Creatinine, µmol/L	105.50 [84.25–147.25]	105.00 [84.00–145.00]	112.00 [86.00–149.00]	.981
eGFR (MDRD), mL/min/1.73 m^2^	47.39 [26.14–62.99]	51.61 [28.65–63.64]	45.46 [26.05–51.04]	.670
PNI	34.25 ± 6.69	34.33 ± 6.71	33.90 ± 6.72	.779
Antiplatelet therapy, *n* (%)	108 (90.8)	87 (90.6)	21 (91.3)	1.000
Anticoagulant therapy, *n* (%)	59 (49.6)	47 (49.0)	12 (52.2)	.964
Lipid-lowering therapy, *n* (%)	93 (78.2)	75 (78.1)	18 (78.3)	1.000
LV ejection fraction, %	28.04 ± 7.41	28.19 ± 7.60	27.42 ± 6.63	.601
LA diameter, cm	4.32 ± .74	4.32 ± .78	4.30 ± .61	.869
P-wave duration, ms	126.56 ± 18.17	126.17 ± 18.68	128.19 ± 16.08	.574
LA reservoir strain, %	14.51 ± 8.31	14.64 ± 8.48	13.99 ± 7.67	.691
LV GLS, %	−10.09 ± 2.59	−9.95 ± 2.53	−10.74 ± 2.85	.148
CHA_2_DS_2_-VASc score	3.48 ± 1.36	3.17 ± 1.24	4.72 ± 1.11	<.001

CRT, cardiac resynchronization therapy; eGFR, estimated glomerular filtration rate; ICD, implantable cardioverter-defibrillator; IQR, interquartile range; LA, left atrial; LASr, LA reservoir strain; LV, left ventricular; MDRD, Modification of Diet in Renal Disease; NLR, neutrophil-to-lymphocyte ratio; PNI, prognostic nutritional index; GLS, global longitudinal strain.

### Electrocardiographic and echocardiographic markers

Univariable linear regression analyses are presented in Supplementary Table S2 and Supplementary Figure S5. Higher FIND-AF score was significantly associated with lower left atrial conduit strain (*β* −.54, 95% CI −.98 to −.09; *P* = .019).

### Clinical outcomes according to FIND-AF risk category

**Figure 1:**
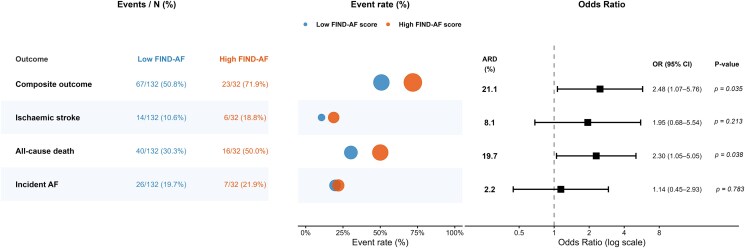
Clinical outcomes according to FIND-AF risk category. Forest plot comparing clinical outcomes between low- and high-risk FIND-AF groups. The composite outcome included incident atrial fibrillation, ischaemic stroke, and all-cause mortality

The primary composite outcome occurred in 23/32 (71.9%) high-risk and 67/132 (50.8%) low-risk patients (OR 2.48, 95% CI 1.07–5.76; *P* = .035) (*Figure 1*; Supplementary Table S3). Absolute risk differences for the composite outcome, all-cause mortality, and ischaemic stroke were 21.1%, 19.7%, and 8.1%, respectively. Of secondary outcomes, high-risk patients also had higher odds of all-cause mortality (16/32 [50.0%] vs 40/132 [30.3%]; OR 2.30, 95% CI 1.05–5.05; *P* = .038). High FIND-AF risk remained associated with the composite outcome after adjustment for AtCM severity as a three-level factor (adjusted OR 2.70, 95% CI 1.14–6.40; *P* = .024). Models incorporating age and sex using Firth’s penalized logistic regression attenuated the association of FIND-AF risk with the primary composite outcome (*Table 2*).

**Table 2:** Multivariable logistic regression analyses of clinical outcomes according to high vs low FIND-AF risk category

Outcome	Model 1		Model 2		Model 3	
	OR (95% CI)	*P*-value	OR (95% CI)	*P*-value	OR (95% CI)	*P*-value
**Composite outcome**	2.48 (1.07–5.76)	.035	2.70 (1.14–6.40)	.024	1.02 (.37–2.90)	.974
**All-cause mortality**	2.30 (1.05–5.05)	.038	2.45 (1.10–5.47)	.028	1.03 (.39–2.71)	.952
**Ischaemic stroke**	1.95 (.68–5.54)	.213	1.89 (.66–5.43)	.239	.86 (.22–3.16)	.822
**Incident AF**	1.14 (.45–2.93)	.783	1.22 (.46–3.20)	.689	.58 (.18–1.74)	.335

Model 1 is unadjusted.

Model 2 is adjusted for AtCM severity as a three-level factor.

Model 3 is adjusted for AtCM severity, age, and sex.

Composite outcome includes incident AF, ischaemic stroke, or all-cause mortality.

### Incremental prognostic value beyond CHA_2_DS_2_-VASc score

**Figure 2:**
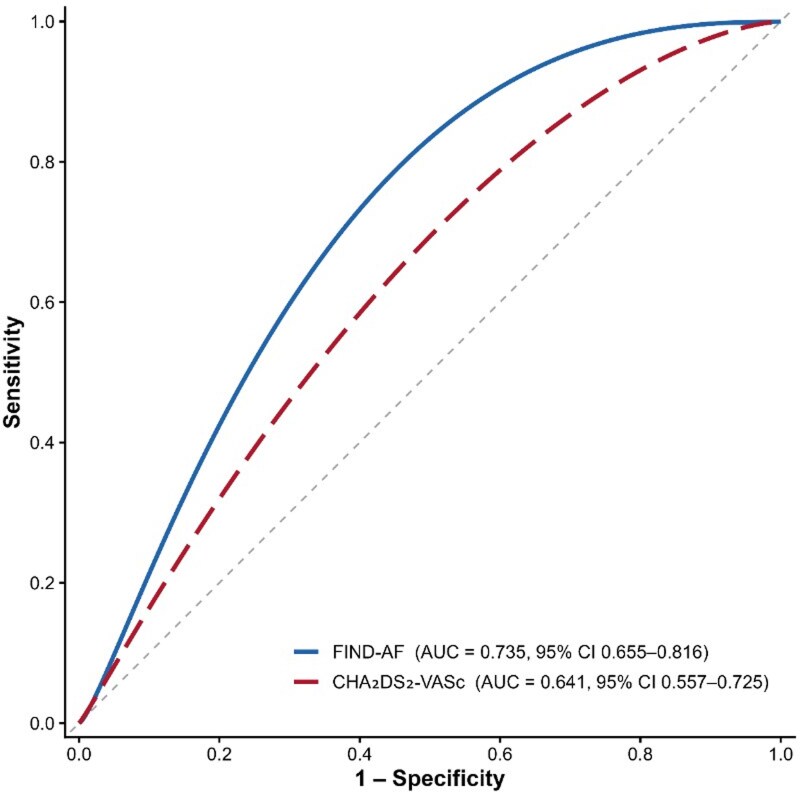
Comparison of discriminative performance between FIND-AF and CHA_2_DS_2_-VASc for the composite outcome. Receiver operating characteristic curves comparing the continuous FIND-AF score with CHA_2_DS_2_-VASc for prediction of the composite outcome, defined as incident atrial fibrillation, ischaemic stroke, or all-cause mortality

FIND-AF showed superior discrimination for the primary composite outcome (AUC .735 vs .641; DeLong *P* = .007) and stroke (AUC .700 vs .575; DeLong *P* = .008) compared with the CHA_2_DS_2_-VASc score (*Figure 2*, Supplementary Table S4). Stratified by CHA_2_DS_2_-VASc score, the prediction performance for the composite outcome for FIND-AF was highest for patients with CHA_2_DS_2_-VASc scores between 1 and 4 (Supplementary Table S5).

### Sensitivity analyses

Using the 2025 clinical consensus statement of the Heart Failure Association of the ESC functional AtCM threshold of LA reservoir strain <23%, the cohort expanded to 191 patients (low risk *n* = 158; high risk *n* = 33). The association between high FIND-AF risk and the composite outcome remained consistent (24/33 [72.7%] vs 80/158 [50.6%]; OR 2.60, 95% CI 1.14–5.95; *P* = .024), as did the association with all-cause mortality (OR 2.36, 95% CI 1.10–5.06; *P* = .027). After exclusion of 59 patients receiving anticoagulant therapy at baseline, 105 patients remained (low risk *n* = 85; high risk *n* = 20). The composite outcome association was directionally consistent but with wider confidence intervals (13/20 [65.0%] vs 38/85 [44.7%]; OR 2.30, 95% CI .83–6.33; *P* = .108). Full sensitivity analysis results are shown in Supplementary Table S6, Panels A and B.

## Discussion

This study examined the association of a scalable data biomarker, FIND-AF, with prognosis in patients with HFrEF and AtCM without known AF. High FIND-AF risk in this cohort was associated with a higher incidence of the primary composite outcome. FIND-AF showed superior prediction to the CHA_2_DS_2_-VASc score for the primary composite outcome.

### Comparison to prior studies

These findings extend prior observations derived from CMR imaging, which demonstrated that higher FIND-AF risk in patients with HFrEF without diagnosed AF corresponds to higher indexed left atrial volume, higher native T1 signal, and higher extracellular volume.^9^ Furthermore, in a non-heart failure population, patients with high FIND-AF risk but without diagnosed AF were found to have comparable levels of atrial fibrosis to those with diagnosed AF.^12^ In the present study, high FIND-AF risk was associated with lower left atrial conduit strain, reflecting reduced compliance and impaired atrioventricular coupling. In totality, these data indicate that high FIND-AF risk identifies patients with HF with atrial remodelling and at risk of adverse events in the absence of diagnosed AF.

### Atrial cardiomyopathy in heart failure with reduced ejection fraction as an actionable treatment target

Atrial cardiomyopathy is defined by the Heart Failure Association of the European Society of Cardiology as electrical and mechanical dysfunction of the atria with the potential to produce clinical consequences. Thus, AtCM may be a specific clinical entity that can be targeted to prevent adverse events. It is hypothesized that aggressive control of risk factors or comorbidities, anti-remodelling treatments (such as neprilysin or sodium–glucose co-transporter 2 inhibitors), and pharmacological agents with anti-fibrotic properties (such as sodium–glucose co-transporter 2 inhibitors and glucagon-like peptide-1 receptor agonists) may delay progression of, or even reverse, atrial cardiomyopathy.^4,15,16^

### Research and clinical implications

Our data show that patients with HFrEF and AtCM have a poor prognosis. Identifying high-risk participants would allow for more intensive monitoring, follow-up and a clear focus on targeting comorbidities and underlying risk factors.^17,18^ There is an absence of a risk score for prognosis in patients with HFrEF and AtCM, and this study provides hypothesis-generating data that FIND-AF is associated with adverse prognosis in this high-risk subgroup. Development of novel models and external validation in larger cohorts are required as age and sex-adjusted analyses attenuated the association of high FIND-AF risk and the primary composite outcome.

Incident AF events were obtained from clinical diagnostic records rather than continuous rhythm monitoring, which may underestimate paroxysmal or asymptomatic AF, and given the high rate of stroke in patients with HFrEF and AtCM, prospective studies may evaluate whether intensive ECG monitoring, earlier clinical follow-up, and targeted comorbidity management, or anticoagulation can improve outcomes in this high-risk group.

This study has several limitations. First, it was observational in nature, and causality cannot be inferred. Second, because only patients with digitally stored XML electrocardiograms were included, the analytic cohort may not represent a fully consecutive series of all heart failure admissions. Third, the modest sample size limited statistical power for multivariable and subgroup means that these results should be considered exploratory. Fourth, individual-level time-to-event and censoring data were not available; therefore, Cox regression and Kaplan–Meier analyses could not be performed, and the current findings should be interpreted as logistic associations rather than time-to-event estimates. Fifth, hospitalization for heart failure was not captured as an endpoint in the current dataset, but it is clinically important and should be incorporated in future analyses and studies. Sixth, although we assessed structural, functional, and electrical markers of AtCM using readily available echocardiography and ECG, the lack of CMR imaging precluded evaluation of atrial fibrosis and detailed tissue characterization. Finally, baseline anticoagulant use was common; although sensitivity analysis was conducted, residual confounding by unrecognized prior paroxysmal AF cannot be completely excluded.

## Conclusion

High FIND-AF risk is associated with adverse prognosis in a cohort of patients with HFrEF and AtCM but without baseline AF. These exploratory data require external validation and a prospective study.

## Supplementary Material

xvag144_Supplementary_Data

## Declarations

### Data Availability

The anonymized datasets are publicly documented in a dissertation accessible via ProQuest (Tse G. Atrial cardiomyopathy and heart failure, 2023; https://www.proquest.com/docview/3252768154).

### Pre-registered Clinical Trial Number

None supplied.

### Transparency

The lead author affirms that the manuscript is an honest, accurate, and transparent account of the study being reported; that no important aspects of the study have been omitted; and that any discrepancies from the study as planned (and, if relevant, registered) have been explained

## References

[1] Carluccio E, Biagioli P, Mengoni A, Francesca Cerasa M, Lauciello R, Zuchi C, et al Left atrial reservoir function and outcome in heart failure with reduced ejection fraction. Circ Cardiovasc Imaging 2018;11:e007696. 10.1161/CIRCIMAGING.118.007696 30571318

[2] Özbek BT, Modin D, Sengeløv M, Jørgensen PG, Bruun NE, Fritz-Hansen T, et al Left atrial strain and all-cause mortality in patients with heart failure with reduced ejection fraction: a retrospective cohort study. BMJ Open 2026;16:e107569. 10.1136/bmjopen-2025-107569 PMC12931534 41730559

[3] Bo K, Gao Y, Zhou Z, Gao X, Liu T, Zhang H, et al Incremental prognostic value of left atrial strain in patients with heart failure. ESC Heart Fail 2022;9:3942–53. 10.1002/ehf2.14106 35950517 PMC9773762

[4] Weerts J, Țica O, Aranyo J, Basile C, Borizanova-Petkova A, Borovac JA, et al Atrial cardiomyopathy: from healthy atria to atrial failure. A clinical consensus statement of the Heart Failure Association of the ESC. Eur J Heart Fail 2025;27:2173–94. 10.1002/ejhf.3782 40763073 PMC12765236

[5] Nadarajah R, Alsaeed E, Hurdus B, Aktaa S, Hogg D, Bates MGD, et al Prediction of incident atrial fibrillation in community-based electronic health records: a systematic review with meta-analysis. Heart 2022;108:1020–9. 10.1136/heartjnl-2021-320036 34607811 PMC9209680

[6] Nadarajah R, Wahab A, Reynolds C, Raveendra K, Askham D, Dawson R, et al Future Innovations in Novel Detection for Atrial Fibrillation (FIND-AF): pilot study of an electronic health record machine learning algorithm-guided intervention to identify undiagnosed atrial fibrillation. Open Heart 2023;10:e002447. 10.1136/openhrt-2023-002447 37777255 PMC10546147

[7] Nadarajah R, Wu J, Hogg D, Raveendra K, Nakao YM, Nakao K, et al Prediction of short-term atrial fibrillation risk using primary care electronic health records. Heart 2023;109:1072–9. 10.1136/heartjnl-2022-322076 36759177 PMC10359547

[8] Wu J, Nadarajah R, Nakao YM, Nakao K, Arbel R, Haim M, et al Risk calculator for incident atrial fibrillation across a range of prediction horizons. Am Heart J 2024;272:1–10. 10.1016/j.ahj.2024.03.001 38458372

[9] Helbitz A, Nadarajah R, Mu L, Larvin H, Ismail H, Wahab A, et al Identification, characterisation and outcomes of pre-atrial fibrillation in heart failure with reduced ejection fraction. ESC Heart Fail 2025;12:3688–96. 10.1002/ehf2.15347 40528830 PMC12450782

[10] Tse G, Zhou J, Woo SWD, Ko CH, Lai RWC, Liu T, et al Multi-modality machine learning approach for risk stratification in heart failure with left ventricular ejection fraction ≤ 45. ESC Heart Fail 2020;7:3716–25. 10.1002/ehf2.12929 33094925 PMC7754744

[11] Zhou J, Li A, Tan M, Lam MCY, Hung LT, Siu RWH, et al P-wave durations from automated electrocardiogram analysis to predict atrial fibrillation and mortality in heart failure. ESC Heart Fail 2023;10:872–83. 10.1002/ehf2.14230 36461637 PMC10053164

[12] Tse G, Zhou J, Lee S, Wong WT, Li X, Liu T, et al Relationship between angiotensin-converting enzyme inhibitors or angiotensin receptor blockers and COVID-19 incidence or severe disease. J Hypertens 2021;39:1717–24. 10.1097/HJH.0000000000002866 34188006

[13] Chan JSK, Zhou J, Lee S, Li A, Tan M, Leung KSK, et al Fragmented QRS is independently predictive of long-term adverse clinical outcomes in Asian patients hospitalized for heart failure: a retrospective cohort study. Front Cardiovasc Med 2021;8:738417. 10.3389/fcvm.2021.738417 34859066 PMC8631899

[14] Lang RM, Badano LP, Mor-Avi V, Afilalo J, Armstrong A, Ernande L, et al Recommendations for cardiac chamber quantification by echocardiography in adults: an update from the American Society of Echocardiography and the European Association of Cardiovascular Imaging. J Am Soc Echocardiogr 2015;28:1–39.e14. 10.1016/j.echo.2014.10.003 25559473

[15] Wahab A, Nadarajah R, Tomoaia R, Javed W, Reynolds C, Bennet S, et al Cardiac magnetic resonance imaging-derived atrial fibrosis in patients with pre-atrial fibrillation. Open Heart 2025;12:e003747. 10.1136/openhrt-2025-003747 41314690 PMC12666032

[16] Pallisgaard J, Greve AM, Lock-Hansen M, Thune JJ, Fosboel EL, Devereux RB, et al Atrial fibrillation onset before heart failure or vice versa: what is worst? A nationwide register study. Europace 2023;25:283–90. 10.1093/europace/euac186 36349557 PMC9935045

[17] McDonagh TA, Metra M, Adamo M, Gardner RS, Baumbach A, Böhm M, et al 2021 ESC Guidelines for the diagnosis and treatment of acute and chronic heart failure. Eur Heart J 2021;42:3599–726. 10.1093/eurheartj/ehab368 34447992

[18] Van Gelder IC, Rienstra M, Bunting KV, Casado-Arroyo R, Caso V, Crijns HJGM, et al 2024 ESC Guidelines for the management of atrial fibrillation developed in collaboration with the European Association for Cardio-Thoracic Surgery (EACTS). Eur Heart J 2024;45:3314–414. 10.1093/eurheartj/ehae176 39210723

