# Supplementary material for: Stratifying prognosis in heart failure patients with reduced ejection fraction and atrial cardiomyopathy
Source: ESC Heart Fail. 2026 May 21;13(3):xvag144. doi: 10.1093/eschf/xvag144 (PMC13256026; doi:10.1093/eschf/xvag144)

**Supplementary Appendix**

Stratifying prognosis in heart failure patients with reduced ejection fraction and atrial cardiomyopathy

Jung Chi Hsu, Umbreen Nadeem, Khalid Kazi, Chris Hayward, Lan Mu, Wing Tak Wong, Gregory Y H Lip, Mark C Petrie, Dipak Kotecha, A John Camm, Jianhua Wu, Gary Tse, Chris P Gale, Ramesh Nadarajah

Supplementary results……………………………………………………………..…………..2

Supplementary Table 1. Operational Definition of Atrial Cardiomyopathy Assessment…………………………2

Supplementary Table 2. Univariable linear regression of FIND-AF score with echocardiographic and electrocardiographic parameters among HFrEF patients with atrial cardiomyopathy…………………………….3

Supplementary Table 3. Incidence Rates and Odds Ratios of Clinical Outcomes by FIND-AF Risk category………………..………………………………………………….…………………………………...…..4

Supplementary Table 4. Predictive Performance of FIND-AF Score versus CHA₂DS₂-VASc score………….....5

Supplementary Table 5. FIND-AF score AUC for Clinical Outcomes Stratified by CHA₂DS₂-VASc score…….6

Supplementary Table 6. Sensitivity Analyses: Association of FIND-AF Risk with Clinical Outcomes…………7

Figure S1. Sex-based distribution of FIND-AF scores……………………………………………………...….....8

Figure S2. Study flow diagram ……………………………………………………………………………...…….9

Figure S3. Distribution of atrial cardiomyopathy severity in the overall study population……………………...10

Figure S4. Prevalence of Atrial Cardiomyopathy Severity and Pathophysiological Domains Stratified by FIND-AF Risk Group…………………………………………………………………………………..………...……..11

Figure S5. Univariable linear regression between FIND-AF score and echocardiographic and electrocardiographic parameters …………………………………………………………………………………12

# Supplementary Results

**Supplementary Table 1. Operational Definition of Atrial Cardiomyopathy Assessment**

Atrial cardiomyopathy was operationalized using a pragmatic multi-domain framework based on the 2025 clinical consensus statement of the Heart Failure Association of the ESC on atrial cardiomyopathy. Each pathophysiological domain (structural, functional, electrical) contributes a maximum of 1 point. The total AtCM score (range 0–3) was categorized as: 0 = no AtCM, 1 = mild, 2 = moderate, 3 = severe.

| **Domain** | **Diagnostic criteria**  **(any one fulfils the domain)** | **Points** | **Source/rationale** |
| --- | --- | --- | --- |
| **Structural** | Sex-specific LA enlargement: LA anteroposterior diameter ≥4.0 cm in men; ≥3.8 cm in women | 1 | Based on the 2025 clinical consensus statement of the Heart Failure Association of the ESC AtCM consensus and ASE/EACVI chamber quantification reference limits. LA anteroposterior diameter was used because LA volume was not consistently available. |
| **Functional** | LA reservoir strain <15% in the main analysis; LA reservoir strain <23% in the 2025 clinical consensus statement of the Heart Failure Association of the ESC sensitivity analysis | 1 | The <15% threshold was selected as a conservative HFrEF-specific threshold based on Carluccio et al.; the <23% threshold was used according to the 2025 clinical consensus statement of the Heart Failure Association of the ESC AtCM consensus. |
| **Electrical** | consensus-based AtCM P-wave score: advanced inter-atrial block = 2 points; partial inter-atrial block, P-wave duration ≥120 ms, or P-wave terminal force in V1 ≥40 ms·mm = 1 point each. The electrical domain was considered positive if the score was ≥1. | 1 | Based on the consensus-based AtCM P-wave score as an ECG-based marker of atrial electrical dysfunction. |

**Abbreviations:** AtCM, atrial cardiomyopathy; LAD, left atrial diameter; PTFV1, P-wave terminal force in V1.

**Clinical Example:**

**A male patient with HFrEF presents with a left atrial diameter of 42 mm (Structural point = 1), an LA reservoir strain of 12% (Functional point = 1), and an advanced inter-atrial block on ECG (P-wave score = 2; Electrical point = 1).**

**Result:**

**The patient is assigned 1 point for each of the three domains, yielding a total AtCM score of 3. According to our framework, this is categorized as Severe Atrial Cardiomyopathy, indicating a high-risk atrial substrate even in the absence of documented atrial fibrillation.**

**Supplementary Table 2.** **Univariable linear regression of FIND-AF score with echocardiographic and electrocardiographic parameters among HFrEF patients with atrial cardiomyopathy**

| **Variable** | **β (95% CI)** | **P-value** |
| --- | --- | --- |
| **Left atrial diameter (cm)** | −0.02 (−0.10 to 0.05) | 0.528 |
| **LV ejection fraction (%)** | 0.10 (−0.64 to 0.85) | 0.785 |
| **P-wave duration (ms)** | 0.71 (−1.13 to 2.56) | 0.446 |
| **P-wave dispersion (ms)** | 0.72 (−1.12 to 2.55) | 0.442 |
| **P-wave terminal force in V1** | 1.62 (−2.96 to 6.19) | 0.486 |
| **LA reservoir strain** | −0.21 (−1.06 to 0.65) | 0.631 |
| **LA contractile strain** | 0.12 (−0.56 to 0.81) | 0.721 |
| **LA conduit strain** | −0.54 (−0.98 to −0.09) | 0.019 |
| **LV global longitudinal strain** | −0.12 (−0.40 to 0.16) | 0.406 |
| **Average ventricular GLS** | -0.15 (-0.46–0.16) | 0.337 |

FIND-AF score was natural-log transformed prior to regression because of its right-skewed distribution. β represents the change in the dependent variable per 1-unit increase in the natural log of the FIND-AF score.

Abbreviations: CI, confidence interval; GLS, global longitudinal strain; LA, left atrial; LV, left ventricular.

**Supplementary Table 3. Incidence Rates and Odds Ratios of Clinical Outcomes by FIND-AF Risk category**

| **Outcome** | **High FIND-AF (n=32)** | **Low FIND-AF (n=132)** | **ARD** | **OR (95% CI)** | **P-value** |
| --- | --- | --- | --- | --- | --- |
| **Composite outcome** | 23/32 (71.9%) | 67/132 (50.8%) | 21.1% | 2.48 (1.07–5.76) | 0.035 |
| **All-cause mortality** | 16/32 (50.0%) | 40/132 (30.3%) | 19.7% | 2.30 (1.05–5.05) | 0.038 |
| **Ischaemic stroke** | 6/32 (18.8%) | 14/132 (10.6%) | 8.1% | 1.95 (0.68–5.54) | 0.213 |
| **Incident AF** | 7/32 (21.9%) | 26/132 (19.7%) | 2.2% | 1.14 (0.45–2.93) | 0.783 |

ARD = absolute risk difference (high-risk minus low-risk). OR with Wald 95% CI from unadjusted logistic regression. Composite outcome includes incident AF, ischaemic stroke, or all-cause mortality.

**Supplementary Table 4. Predictive Performance of FIND-AF Score versus CHA₂DS₂-VASc score**

| **Outcome** | **CHA₂DS₂-VASc**  **OR (95% CI)** | **P-value** | **CHA₂DS₂-VASc AUC (95% CI)** | **FIND-AF AUC (95% CI)** | **DeLong P-value** |
| --- | --- | --- | --- | --- | --- |
| **Composite outcome** | 1.44 (1.13–1.84) | 0.003 | 0.641 (0.557–0.725) | 0.735 (0.655–0.816) | 0.007 |
| **All-cause mortality** | 1.65 (1.27–2.15) | <0.001 | 0.695 (0.612–0.778) | 0.692 (0.610–0.775) | 0.942 |
| **Ischaemic stroke** | 1.18 (0.84–1.66) | 0.337 | 0.575 (0.459–0.691) | 0.700 (0.589–0.812) | 0.008 |
| **Incident AF** | 1.18 (0.90–1.57) | 0.236 | 0.563 (0.454–0.672) | 0.630 (0.528–0.733) | 0.193 |

ORs are per 1-unit increase in the CHA₂DS₂-VASc score with Wald 95% CIs from logistic regression. AUCs are with DeLong 95% CIs. DeLong P-value compares FIND-AF and CHA₂DS₂-VASc AUCs.

**Supplementary Table 5. FIND-AF score AUC for Clinical Outcomes Stratified by CHA₂DS₂-VASc score**

| **CHA₂DS₂-VASc** | **N** | **Comp. events** | **Composite AUC (95% CI)** | **AF events** | **AF AUC (95% CI)** | **Stroke ev.** | **Stroke AUC (95% CI)** | **Mortality ev.** | **Mortality AUC (95% CI)** |
| --- | --- | --- | --- | --- | --- | --- | --- | --- | --- |
| 1 | 8 | 2 | 0.833  (0.507–1.000) | 1 | 0.786 (NA–NA) | 0 | N/A | 1 | 0.786  (NA–NA) |
| 2 | 38 | 16 | 0.685  (0.502–0.868) | 6 | 0.669  (0.417–0.921) | 3 | 0.457  (0.018–0.896) | 7 | 0.647  (0.398–0.897) |
| 3 | 39 | 19 | 0.801  (0.664–0.939) | 9 | 0.781  (0.603–0.960) | 5 | 0.876  (0.745–1.000) | 7 | 0.583  (0.345–0.821) |
| 4 | 42 | 26 | 0.707  (0.536–0.877) | 6 | 0.442  (0.114–0.770) | 7 | 0.784  (0.640–0.927) | 20 | 0.644  (0.471–0.818) |
| 5 | 21 | 18 | 0.444  (0.000–0.957) | 7 | 0.541  (0.265–0.816) | 4 | 0.618 (  0.264–0.972) | 15 | 0.378  (0.046–0.709) |
| 6 | 16 | 9 | 0.373  (0.048–0.698) | 4 | 0.302  (0.000–0.645) | 1 | 0.267 (NA–NA) | 6 | 0.467  (0.150–0.784) |

AUCs computed using FIND-AF score within each CHA₂DS₂-VASc stratum. NA = CI could not be estimated (single event); N/A = AUC not estimable (no events in stratum).

**Supplementary Table 6. Sensitivity Analyses: Association of FIND-AF Risk with Clinical Outcomes**

**Panel A. Using 2025 clinical consensus statement of the Heart Failure Association of the ESC functional AtCM threshold (LA reservoir strain <23%); cohort N=191**

| **Outcome** | **Low FIND-AF (n=158)** | **High FIND-AF (n=33)** | **OR (95% CI)** | **P-value** |
| --- | --- | --- | --- | --- |
| **Composite outcome** | **80/158 (50.6%)** | **24/33 (72.7%)** | **2.60 (1.14–5.95)** | **0.024** |
| **All-cause mortality** | **49/158 (31.0%)** | **17/33 (51.5%)** | **2.36 (1.10–5.06)** | **0.027** |
| **Ischaemic stroke** | **15/158 (9.5%)** | **6/33 (18.2%)** | **2.12 (0.75–5.95)** | **0.154** |
| **Incident AF** | **29/158 (18.4%)** | **7/33 (21.2%)** | **1.20 (0.47–3.03)** | **0.703** |

**Panel B. After excluding patients on baseline anticoagulant therapy; cohort N=105**

| **Outcome** | **Low FIND-AF (n=85)** | **High FIND-AF (n=20)** | **OR (95% CI)** | **P-value** |
| --- | --- | --- | --- | --- |
| **Composite outcome** | **38/85 (44.7%)** | **13/20 (65.0%)** | **2.30 (0.83–6.33)** | **0.108** |
| **All-cause mortality** | **23/85 (27.1%)** | **7/20 (35.0%)** | **1.45 (0.52–4.09)** | **0.481** |
| **Ischaemic stroke** | **11/85 (12.9%)** | **4/20 (20.0%)** | **1.68 (0.47–5.96)** | **0.421** |
| **Incident AF** | **9/85 (10.6%)** | **4/20 (20.0%)** | **2.11 (0.58–7.71)** | **0.258** |

**ORs reported with Wald 95% CIs from unadjusted logistic regression. Composite outcome includes incident AF, ischaemic stroke, or all-cause mortality.**

## Figure S1. Distribution of FIND-AF Scores Stratified by Sex. (A) The overall distribution of FIND-AF scores in the study population is markedly right-skewed (skewness = 2.06). The red line represents the kernel density estimate. (B) Sex-stratified density plots show that female patients (red) have a broader distribution with a heavier right tail than male patients (blue), indicating greater dispersion of higher FIND-AF scores among women.


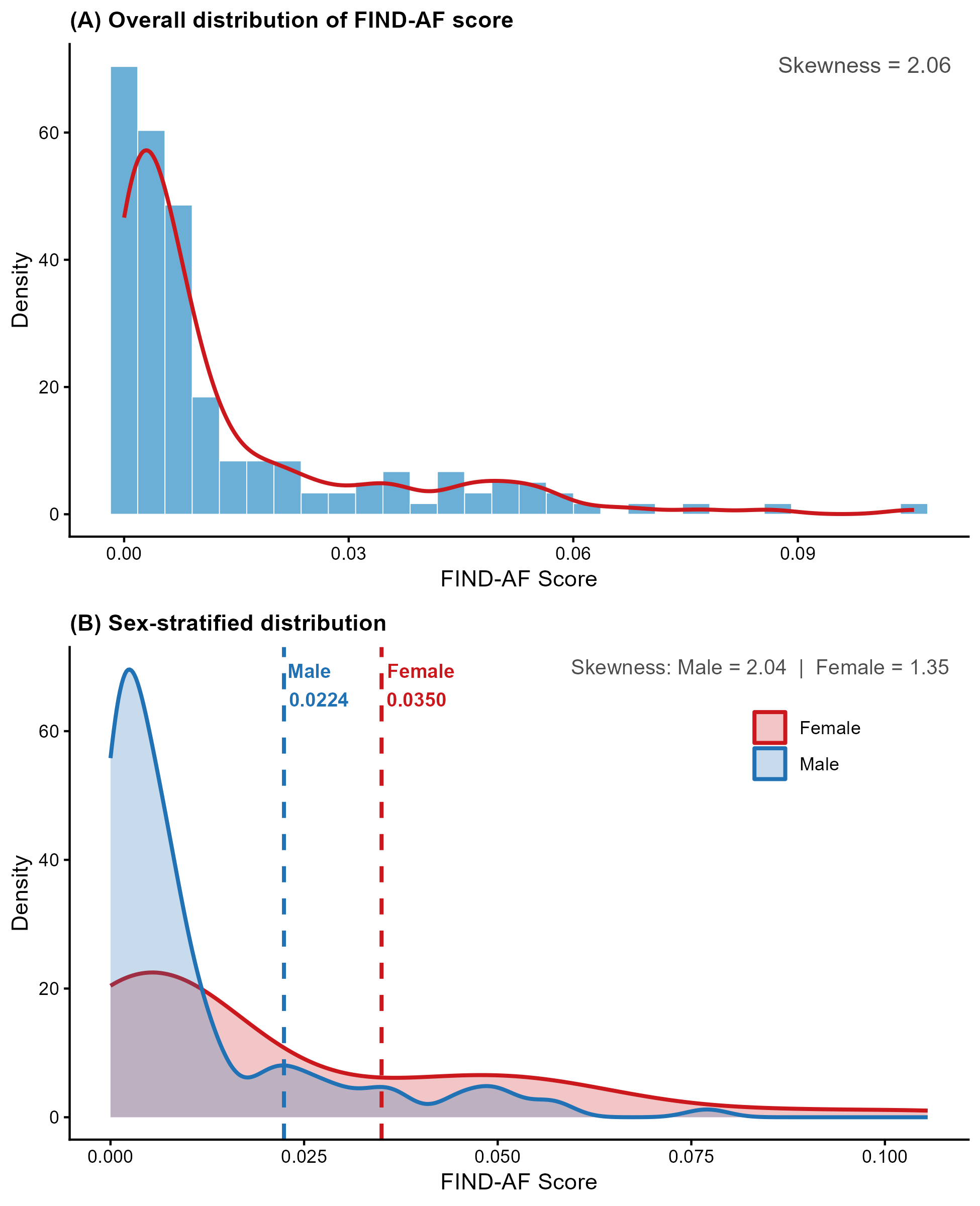


## Figure S2. Study flow diagram


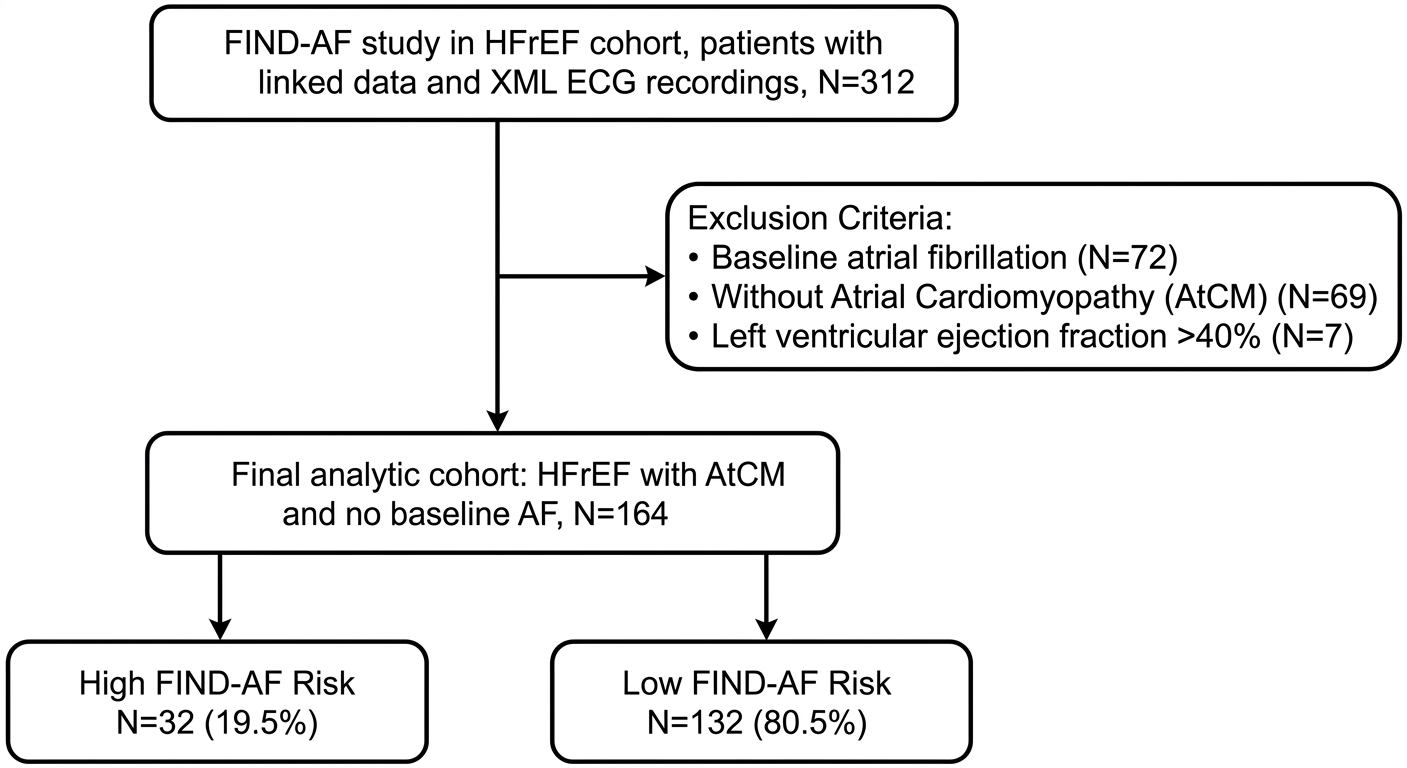


Abbreviations: AF, atrial fibrillation; AtCM, atrial cardiomyopathy; HFrEF, heart failure with reduced ejection fraction.

**Figure S3. Distribution of atrial cardiomyopathy severity in the overall study population**

**
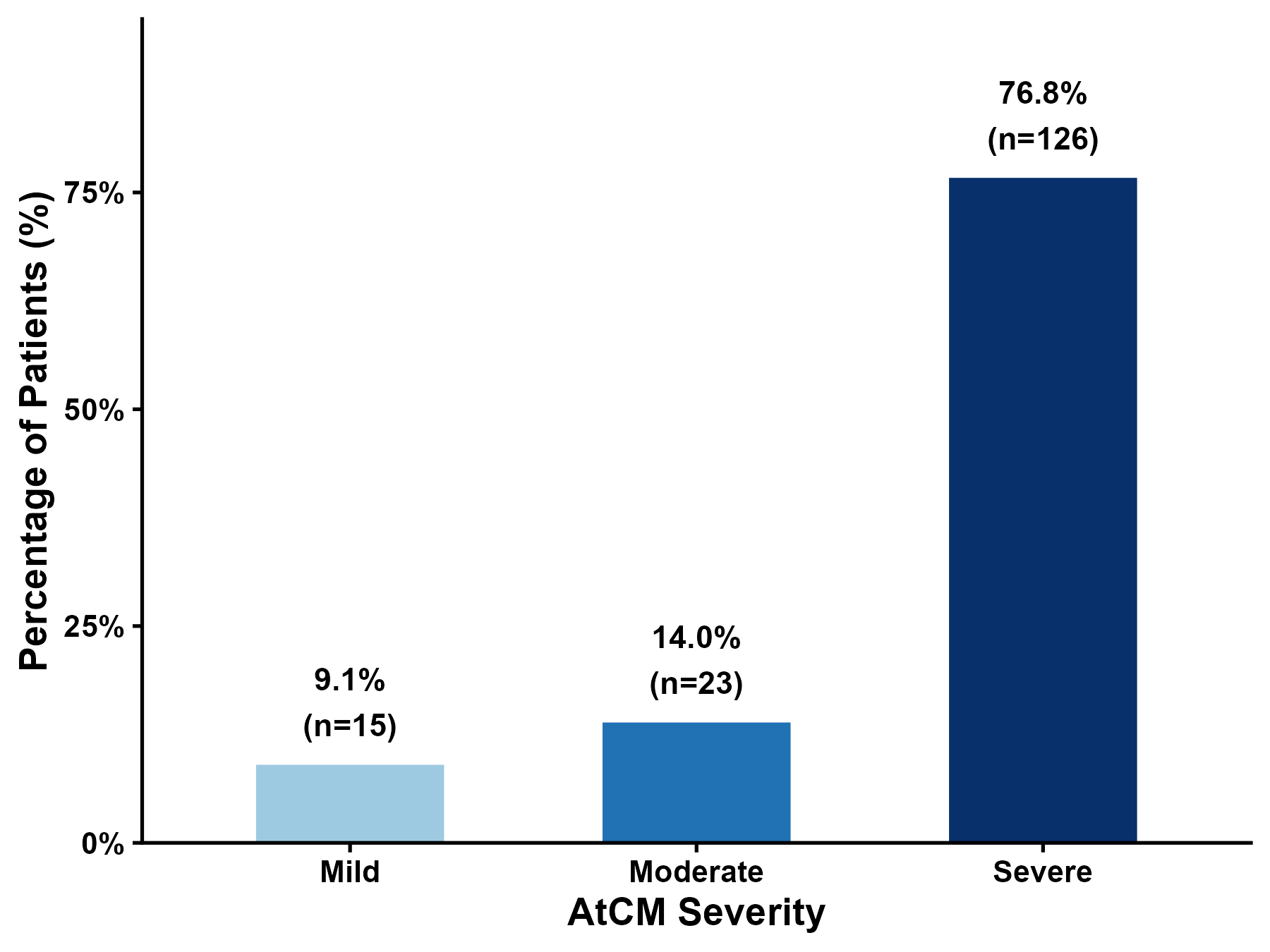
**

Abbreviations: AtCM, atrial cardiomyopathy; HFrEF, heart failure with reduced ejection fraction.

**Figure S4. Prevalence of Atrial Cardiomyopathy Severity and Pathophysiological Domains Stratified by FIND-AF Risk Group**


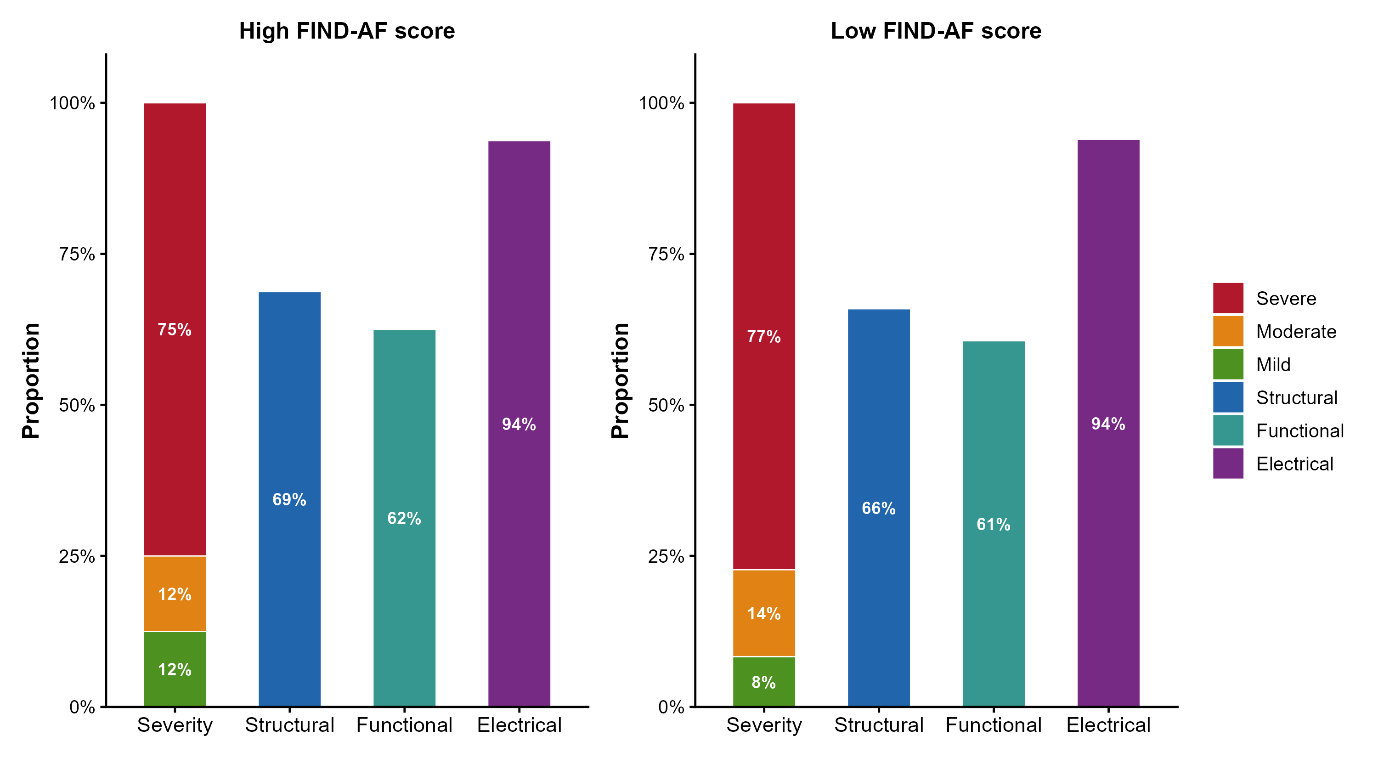


**Figure S5. Univariable linear regression between FIND-AF score and echocardiographic and electrocardiographic parameters**

Higher FIND-AF score was significantly associated with lower LA conduit strain (β=−0.54, 95% CI −0.98 to −0.09; p=0.019), while no significant associations were observed for other echocardiographic or electrocardiographic parameters


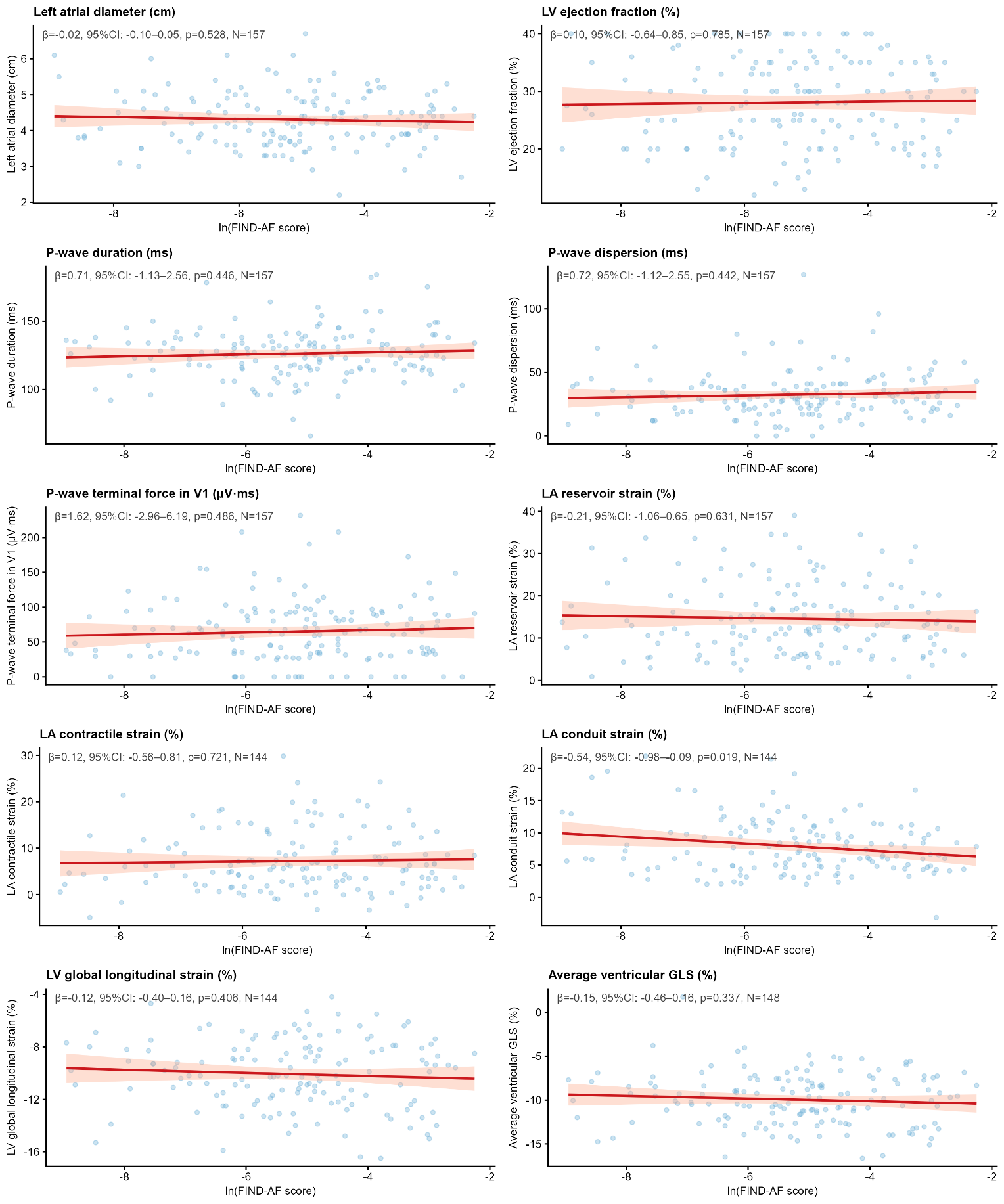

Supplement: xvag144_Supplementary_Data [file xvag144_supplementary_data.docx]
